# Autophagy and Exosome Coordinately Enhance Macrophage M1 Polarization and Recruitment in Influenza A Virus Infection

**DOI:** 10.3389/fimmu.2022.722053

**Published:** 2022-03-17

**Authors:** Chengjie Xia, Weiming Xu, Xin Ai, Yingqi Zhu, Ping Geng, Yijun Niu, Haiyan Zhu, Wei Zhou, Hai Huang, Xunlong Shi

**Affiliations:** ^1^ Department of Biological Medicines & Shanghai Engineering Research Center of Immunotherapeutics, Fudan University School of Pharmacy, Shanghai, China; ^2^ Department of Chemistry, Fudan University, Shanghai, China

**Keywords:** influenza, macrophage, polarization, LC3, CD63

## Abstract

**Background:**

Influenza A virus infection results in viral pneumonia, which is often accompanied by the infiltration and recruitment of macrophages, overactivation of inflammatory responses, and obvious cell autophagy and exosome production. However, little is known about the roles of autophagy and exosome production in these inflammatory responses.

**Methods:**

In this study, multiple methods, such as flow cytometry, real-time quantitative reverse transcription-polymerase chain reaction, immune–fluorescence technology, and western blot, were applied to explore the possible effects of autophagy and exosome production by H1N1-infected host cells.

**Results:**

It was observed that a high number of polarized macrophages (CD11b^+^/F4/80^+^/CD86^+^) were recruited to the lung tissues of infected mice, which could be mimicked by tracking the movement of macrophages to H1N1-infected cells *in vitro* (transwell assays). Furthermore, there was some coordinated upregulation of M1 polarization signs (iNOS/Arg-1 bias) as well as autophagy (LC3) and exosome (CD63) biomarkers in the infected macrophages and epithelial cells. Moreover, exosomes extracted from the supernatant of virus-infected cells were shown to promote the recruitment and polarization of more peritoneal macrophages than the normal group. The fluorescence colocalization of LC3-CD63 and the inhibition of autophagy and exosome signaling pathway further revealed that H1N1 infection seemed to sequentially activate the M1 polarization and recruitment of macrophages *via* autophagy–exosome dependent pathway.

**Conclusion:**

Autophagy and exosome production coordinately enhance the M1 polarization and recruitment of macrophages in influenza virus infection, which also provides potential therapeutic targets.

**Graphical Abstract:**
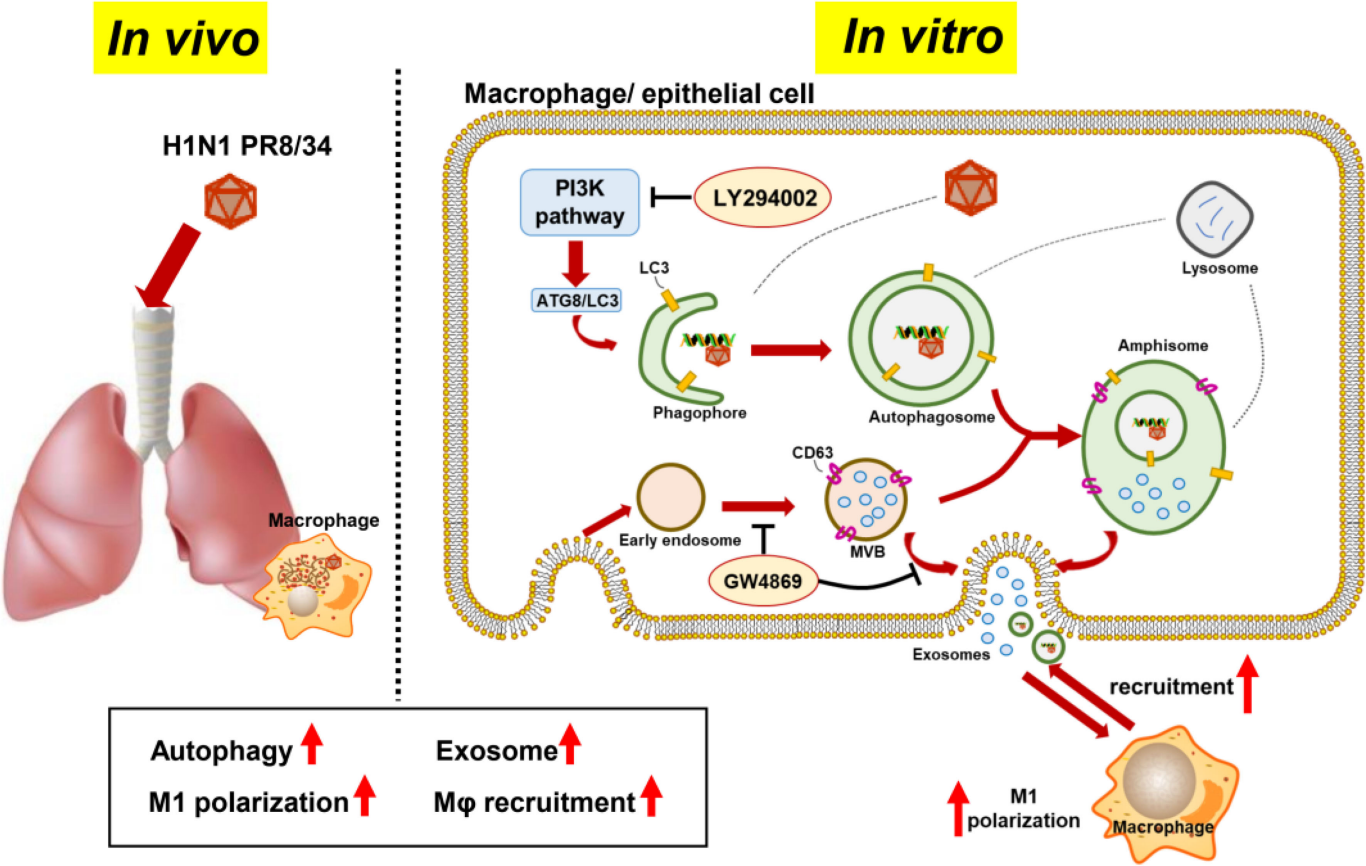
*In vivo* experiment, H1N1 virus infection caused recruitment and M1 polarization of macrophages in the lung, accompanied by the increasement of LC3 and CD63 expression, as autophagy and exosome markers. *In vitro* experiment, H1N1 virus also promoted the formation of autophagosomes and exosomes in macrophages and epithelial cells. Based on the assumption that autophagosomes could fuse with multivesicular bodies (MVBs) to formulate amphisomes, to induce colocalization of LC3 and CD63 in virus-infected cells. Besides, secreted exosomes were found to induce M1 polarization and recruitment of adjacent macrophages. Moreover, LY294002 and GW4869 inhibited recruitment of macrophages via inhibiting formation/maturation of autophagosomes and exosomes in virus-infected cells.

## Introduction

Influenza A viruses (IAVs) are negative-sense, single-stranded, segmented RNA viruses and include several subtypes that are distinguished by the type of hemagglutinin (HA) and neuraminidase (NA) present on the viral surface. IAVs are a leading cause of respiratory infection and an ongoing threat to public health globally. According to the latest World Health Organization report, annual influenza epidemics are estimated to result in approximately 3–5 million cases of severe illness and approximately 290,000–650,000 respiratory deaths worldwide (1). In extreme cases, primary viral pneumonia with rapid progression leads to lung failure and is associated with a high risk of fatal outcomes (2). Currently available vaccinations and antiviral agents are not effective in tackling this problem (3–5). Therefore, novel and effective strategies for the prevention and treatment of IAV infection are the need of the hour.

Overactivation of the host’s innate immune system is a significant factor that causes viral pneumonia (6, 7). Fatal influenza infections activate the innate immune cells of the host, such as macrophages, dendritic cells, and neutrophils. These overactivated immune cells secrete numerous inflammatory cytokines/chemokines, such as IL-6, TNF-α, CXCL1, and CXCL10, which play a crucial role in IAV-induced lung pathology (8–10). Therefore, suppressing these overactivated immune cells could serve as a practical therapeutic approach for viral pneumonia.

Macrophages, which are one of the primary sources of inflammatory cytokines/chemokines (such as TNF-α and IL6), act as critical modulators of IAV disease severity and the development of lethal pulmonary injury (11–13). Shifts in the phenotype of macrophages between the classically activated (M1, proinflammatory) and alternatively activated (M2, anti-inflammatory) types have been recognized as a crucial factor in the initiation, progression, and termination of numerous inflammatory diseases (14–16), especially influenza virus infection (17–19). In our previous study, we found that a TNF-α inhibitor (20) and a cell autophagy inhibitor (21) were capable of protecting against influenza virus infection, which was possibly related to macrophages. However, the underlying linkage among these factors (influenza, macrophage, autophagy) is yet to be elucidated.

This study investigated the possible correlation among macrophage recruitment, M1/M2 polarization, viral replication, autophagy, and exosome production in IAV-infected *in vitro* and *in vivo* experimental models.

## Materials and Methods

### Chemicals and Reagents

Antibodies against mouse CD11b, F4/80, CD86, and CD206 were obtained from BD Biosciences (San Jose, CA, USA). Antibodies against mouse CD63, IL-1β, cleaved IL-1β, caspase-1, and GAPDH were sourced from Affinity Biosciences (Cincinnati, OH, USA). Antibodies against LC3-I/II, p62, and anti-rabbit IgG Fab2 Alexa Fluor^®^ 488 molecular probes were purchased from Santa Cruz Biotechnology (Santa Cruz, CA, USA). Antibodies against CD9 (ab92726) and syntenin (ab19903) were obtained from Abcam (Cambridge, USA). Unless otherwise specified, HRP-conjugated anti-rabbit IgG secondary antibody, FITC-labeled goat anti-mouse IgG (H+L), Cy3-labeled goat anti-rabbit IgG (H+L), and all other chemicals were obtained from Beyotime Biotechnology (Shanghai, China).

### Experimental H1N1 Infection *In Vivo*

Male BALB/c mice (weight: 16-18 g, age: 6-8 weeks) obtained from the Shanghai SLACCAS Laboratory Animal Co., Ltd. (Shanghai, China) were housed under specific pathogen-free (SPF) conditions and provided free access to sterile water and standard mouse chow feed. All experimental protocols were approved by The Animal Experiment Committee of Fudan University (Shanghai, China) (Approval number: 2018-03-WY-SXL-01). All efforts were made to minimize animal pain and discomfort. The number of animals used in the experiment was minimized on a guaranteed basis of sufficient data analysis.

The influenza virus A/FM/1/47 (H1N1) was used for the *in vivo* experiments. This virus strain, isolated from the patients at Fort Monmouth (NJ, USA) during an outbreak in 1947, is a highly virulent, mouse-adapted virus that can cause severe pneumonia, with a high mortality rate in mice. The virus was supplied by the Shanghai Center for Disease Control & Prevention (Shanghai, China) and stored in aliquots at -70°C.

Under isoflurane anesthesia, the experimental mice were infected intranasally (i.n.) with 10LD_50_ (equal to 2.1 × 10^3^ PFU) of influenza virus A/FM/1/47 (H1N1) in a 30-μL-inoculum volume per mouse, and with the normal control mice treated with Dulbecco’s modified Eagle’s medium (DMEM). Three animal experiments are recruited. First, to record the survival rates, the body weight and the survival of mice (n = 10, per group) were recorded daily. Second, the mice (n = 6, per group) on day 4 post-infection were sacrificed. The lung lobes were harvested to calculate the lung/body index, evaluate the lung histopathology score, conduct further experiments (which included quantification of the total protein content, ELISA assay of TNF-α, H&E staining, and immunofluorescence histochemical staining). Third, the mice (n = 6, per group) on day 4 post-infection were anesthetized, and the bronchoalveolar lavage fluid (BALF) was collected to identify the macrophage phenotypes by flow cytometry.

### Lung Histopathology

On day 4 post-infection, the mice (n = 6, per group) were euthanized with 10% phenobarbital in the NaCl solution (250 mg/kg), weighed, and sacrificed. The lung lobes were harvested and weighed to calculate the lung/body index. The right lobes were homogenized in PBS buffer for the quantification of the total protein content and TNF-α by using commercial ELISA kits (BD Biosciences). The left lobes were suspended in PBS-buffered formalin and preserved in paraffin blocks as per the standard procedures. Next, tissue sections (10-µm-thick) were prepared, placed on glass slides, and stained with hematoxylin and eosin (HE) using conventional techniques. The lung histopathology score was calculated according to the histopathological severity of the analyzed sections of each lung (six separate random fields per tissue section). The scores were assigned as follows: normal = 0; minor = 1; mild= 2; intermediate = 3; and severe = 4 (22, 23).

### The Phenotype of Macrophages in the BALF

On day 4 post-infection, the BALF was collected thrice by lavage with 0.5-mL ice-cold PBS from the mice (n = 6, per group). The collected BALF was centrifuged at 700 ×*g* at 4°C for 5 min, and the harvested cells were resuspended in 200-μL PBS. The cells were stained with fluorescently labeled antibodies against the following mouse proteins: CD11b^+^ and F480^+^ (macrophages); CD11b^+^, F480^+^, and CD86^+^ (triple positive, M1 phenotype); and CD11b^+^, F480^+^, and CD206^+^ (triple positive, M2 phenotype). The expression of the member proteins was measured on the BD FACSAria II flow cytometer, and the data were analyzed by the CytExpert software.

### Immunofluorescence Histochemical Staining

The experimental mice (n = 6, per group) on day 4 post-infection were sacrificed, and the lung lobes were harvested to prepare paraffin sections. The paraffin sections of the lung tissues were heated at 65°C for 30 min and washed in dimethyl benzene for 30 min. Then, we used 100%/90%/80%/70% ethanol to hydrate the paraffin sections for 4 min each at room temperature. The paraffin sections were transferred to 10 mM sodium citrate buffer in a 99°C water bath for 20 min, incubated in 3% H_2_O_2_ methanol for 15 min, and blocked with a blocking buffer (Beyotime Biotechnology) for 30 min in a 37°C water bath. The paraffin sections were probed with F4/80 and CD11b antibodies (1:1000, fluorescently labeled) at 4°C overnight and stained with DAPI. The paraffin sections were incubated with antibodies against CD63 and LC3 at 4°C overnight, FITC- or Cy3-labeled secondary antibodies at 37°C for 1 h, and stained with DAPI.

### Experimental H1N1 Infection in Macrophages and Epithelial Cells

For *in vitro* experiments, the influenza A/PR/8/34 TC adapted (H1N1) was obtained from ATCC (VR,1469 AC) and stored in aliquots at -70°C.

Murine and epithelial cells (A549 and BEAS-2B) were obtained from the Cell Bank of Shanghai Institute of Biochemistry and Cell Biology of the Chinese Academy of Sciences (Shanghai, China).

Epithelial cells (A549 or BEAS-2B) were cultured in the DMEM supplemented with 10% (v/v) heat-inactivated fetal bovine serum (FBS), 0.3 mg/mL l-glutamine, 100 U/mL penicillin, and 100 μg/mL streptomycin (Gibco) at 37°C under 5% CO_2_. The cells were cultured to 80% confluence, supplemented with 0.5 μg/mL TPCK-treated trypsin (Sigma-Aldrich, Germany), infected with 10 TCID_50_ of the H1N1 virus for 2 h, and then transferred to a serum-free culture medium.

ANA-1 macrophages and primary peritoneal macrophages (isolated from SPF BALB/c mice) were cultured in the Roswell Park Memorial Institute Medium (RPMI)-1640 media with 10% (v/v) heat-inactivated fetal bovine serum, 0.3 mg/mL l-glutamine, 100 U/mL penicillin, and 100 μg/mL streptomycin (Gibco) at 37°C under 5% CO_2_ and infected similarly.

### Real-Time Quantitative RT-PCR Analysis

According to the manufacturer’s instructions, total RNA was isolated from ANA-1 cells and mouse peritoneal macrophages using TRIzol reagent (Invitrogen, CA, USA) and then converted into cDNA using the Reverse Transcription Kit (Takara, Japan). qRT-PCR was performed using the StepOne Plus RT-PCR System (Applied Biosystems) using the following thermocycling parameters: 94°C for 5 min, followed by 40 cycles of 94°C for 5 s and 60°C for 30 s (20). We normalized the mRNA levels of *iNOS, Arg-1, influenza M gene, IL-1β, IL-13*, and *Tnf-α* to the geometric mean of the *Gapdh* mRNA levels. The primers used in this study are shown in **Table 1**. Each group included at least three independent samples.

**Table 1:** Real-time PCR primers.

Name	Oligo	Primer sequence
influenza A virus M gene	Forward primer	5’-GACCGATCCTGTCACCTCTGAC-3’
	Reverse primer	5’-AGGGCATTCTGGACAAAGCGTCTA-3’
GAPDH	Forward primer	5’-ACCACCATGGAGAAGGCTGG-3’
	Reverse primer	5’-CTCAGTGTAGCCCAGGATGC-3’
iNOS	Forward primer	5’-TCCTGGAGGAAGTGGGCCGAAG -3’
	Reverse primer	5’-CCTCCACGGGCCCGGTACTC -3’
Arg-1	Forward primer	5’-CAGAAGAATGGAAGAGTCAG -3’
	Reverse primer	5’-CAGATATGCAGGGAGTCAC -3’
IL-1β	Forward primer	5’-GCCCATCCTCTGTGACTCAT -3’
	Reverse primer	5’-AGGCCACAGGTATTTTGTCG -3’
IL-13	Forward primer	5’-TGAGCAACATCACACAAGACC-3’
	Reverse primer	5’-GGCCTTGCGGTTACAGAGG-3’
TNF-α	Forward primer	5’-GGAACACGTCGTGGGATAATG-3’
	Reverse primer	5’-GGCAGACTTTGGATGCTTCTT-3’
β-actin	Forward primer	5’-AAGGCCAACCGTGAAAAGAT-3’
	Reverse primer	5’-GTGGTACGACCAGAGGCATAC-3’

### Western Blotting

Total cellular proteins were extracted from the infected primary macrophages and analyzed by Western blotting. Briefly, the cells were washed twice with ice-cold PBS and then lysed with RIPA buffer on an ice bath for 1 h. Equal amounts of total protein of each sample (determined by the BCA assay) were separated by 10% SDS-PAGE and then electrophoretically transferred to the polyvinylidene difluoride (PVDF) membranes (Millipore). The membranes were blocked with the QuickBlock™ Blocking Buffer (Beyotime) and incubated with an appropriate primary antibody (1:1000 dilution) at 4°C overnight. The PVDF membranes were washed thrice times with TBST and incubated with the horseradish peroxidase-conjugated secondary antibody (1:2000 dilution) at 25°C for 1 h. The proteins were visualized using an enhanced chemiluminescence kit (Millipore).

### Macrophage Recruitment (Transwell) Assay

ANA-1 macrophage cells (labeled with GFP lentiviral vectors from Genechem, Shanghai, China) were cultivated in the upper chamber of a 24-well Transwell plate (10^4^ cells/well). ANA-1 or BEAS-2B cells (labeled with mRFP-LC3 lentiviral vectors) or A549 cells were cultivated in the lower chamber (10^5^ cells/mL). The cells in the lower chambers were infected with 10 TCID_50_ of the H1N1 virus for 2 h and then transferred to a fresh culture medium. Macrophage recruitment was evaluated by tracking the movement of GFP^+^ ANA-1 cells under the Leica EL6000 Microscope (Leica Microsystems CMS GmbH).

### Cell Immune-Fluorescence Staining Analysis

The cells were fixed with 4% paraformaldehyde buffer for 30 min, permeabilized with 0.5% Triton X-100 for 20 min, and blocked with blocking buffer (Beyotime) for 30 min at room temperature. The cells were probed with LC3, IL-1β, and CD63 antibodies (1:1000 dilution) at 4°C overnight and subsequently detected with anti-rabbit IgG Fab2 Alexa Fluor^®^ 488 molecular probes (1:2000 dilution). After staining the cell nuclei with DAPI (0.1 µg/mL stock solution) for at least 3 min, the immunofluorescent images were obtained using the Leica EL6000 Microscope (Leica Microsystems).

### Exosome Extraction and Peritoneal Macrophage Recruitment (Transwell) Assay

A549 cells were infected with 10 TCID_50_ of H1N1 virus (A/PR/8/34, ATCC) for 2 h and then transferred to a fresh culture medium. The supernatant of infected or normal A549 cells was collected and centrifuged at 300 ×*g* for 10 min, 2,000 ×*g* for 20 min, and 10,000 ×*g* for 30 min, which contained the conditioned medium after each centrifugation step. The conditioned medium was collected in a new Seal tube and centrifuged for 70 min at 100,000 ×*g*, 4°C with the rotor of P70AT in the Hitachi Himac CP100WX Preparative Ultracentrifuge. The precipitate extracted from 80 mL supernatant was redissolved in a 150-μL fresh culture medium and then purified using 70-nm qEV2 size-exclusion chromatography (SEC) columns (H-wayen Biotechnologies, China).

The International Society for Extracellular Vesicles (ISEV) established Minimal Information for Studies of Extracellular Vesicles (MISEV) guidelines for the analysis of extracellular vesicles (EVs), including exosomes. According to this criterion, we characterized the microscopic appearance, particle size, and specific markers of exosomes (24). The exosome sample was observed under transmission electron microscopy (Hitachi, HT-7700), and the particle diameter and concentration were measured using a particle size analyzer (NanoFCM, N30E). The exosome samples were incubated with FITC-labeled antibodies against CD63 or IgG at 37°C for 30 min and detected using NanoFCM. Other specific markers of exosomes, CD9, and syntenin, were detected by Western blotting.

Each male BALB/c mouse (weight: 16-18 g, age: 6-8 weeks) was intra-peritoneal injected with 4% sodium thioglycolate for 2 mL. After 3 days, primary peritoneal macrophages were isolated and cultivated in the upper chamber of 24-well transwell plates (10^5^ cells/well). The lower rooms were filled with a fresh medium containing exosomes, whose concentration was consistent with that of the original culture supernatant. After 24 h, macrophage recruitment was evaluated by counting the numbers of peritoneal macrophages in the lower chambers under a microscope. The cells in the upper and lower chambers were collected to extract total RNA for qRT-PCR analysis.

### Statistical Analyses

All statistical analyses were performed using GraphPad Prism for Windows (Version 6.0) and presented as mean ± standard deviation (SD). The survival of mice was analyzed by the Gehan–Breslow–Wilcoxon test. Meanwhile, the other experimental data were evaluated by the two-tailed Student’s *t*-test or one-way analysis of variance (ANOVA), followed by Bonferroni’s test. In all cases, probability levels less than 0.05 (*P* < 0.05) were considered to indicate statistical significance.

## Results

### Influenza Virus Infection Caused Massive Recruitment of M1 Macrophages Into the Lung Tissues of Mice

**Figure 1:**
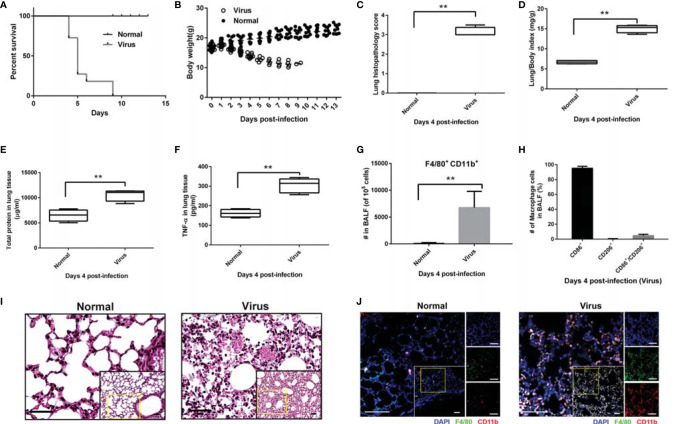
High mortality and macrophage-related overactivation of inflammatory responses in infected mice. Mice (n = 6 or 10, per group) were infected with freshly prepared influenza virus A/FM/1/47 (H1N1) strain. Mice (n = 10, per group) and monitored daily for survival and body weight. For lung histopathology, the mice (n = 6, per group) were euthanized with 10% phenobarbital to harvest the whole lungs on day 4 post-infection. **(A)** Percentage survival. Data below individual survival curves represent the number of survivors/total number of mice. **(B)** Bodyweight. Mice were weighed every day. **(C)** Lung histopathology score. Data were calculated by evaluating the histopathological severity of the analyzed sections of each lung (six separate random fields per tissue section); the scores were assigned as: normal = 0; minor = 1; mild= 2; intermediate = 3; and severe = 4. **(D)** Lung/body index was calculated as follows: whole lung weight (mg)/body weight. **(E, F)** The right lobes of the mice were homogenized to quantify the total protein **(E)** and inflammatory cytokine TNF-α **(F)**. **(G-H)** Flow cytometric analysis of the macrophage number and phenotype in BALF; macrophages (CD11b and F4/80 positive cells), M1 polarized macrophages (CD11b, F4/80 and CD86 triple-positive cells), and M2 polarized macrophages (CD11b, F4/80 and CD206 triple-positive cells). Mean ± SD from at least three independent experiments are shown. **p < 0.01. **(I)** H&E staining of the lung tissues. Scale bar: 40 μm. **(J)** Immunofluorescence histochemical staining of the lung tissues. Scale bar: 50 μm.

As shown in **Figures 1A, B**, the infection with 10 LD_50_ influenza virus A/FM/1/47 caused 100% mortality and approximately 35% body weight loss within 9 days post-infection. Severe viral pneumonia was observed in the infected mice on Day 4 post-infection, which showed that H1N1 virus infection triggered alveolar tissue destruction (*P* < 0.01, **Figure 1C**), pulmonary edema, and hemorrhage (*P* < 0.01, **Figures 1D, E**), accompanied by high levels of the inflammatory cytokine TNF-α (*P* < 0.01, **Figure 1F**).

The results of flow cytometry revealed that these overactivated inflammatory responses correlated positively with the massive macrophage recruitment (nearly 10^5^, CD11b^+^, F4/80^+^, *P* < 0.01) in the BALF of infected mice, of which 95% were of the M1 phenotype (CD11b^+^, F4/80^+^, CD86^+^, triple positive), as shown in **Figures 1G, H**. Meanwhile, few macrophages were detected in the BALF of normal mice. HE and immunofluorescence histochemical staining of lung tissues further confirmed that the H1N1 virus promoted the recruitment of macrophages (**Figures 1I, J**). Thus, the recruitment and M1 polarization of macrophages might be involved in influenza virus-induced lung pathological destruction and inflammatory responses.

### H1N1 Infection Triggered Similar Macrophage Recruitment *In-Vitro*

**Figure 2:**
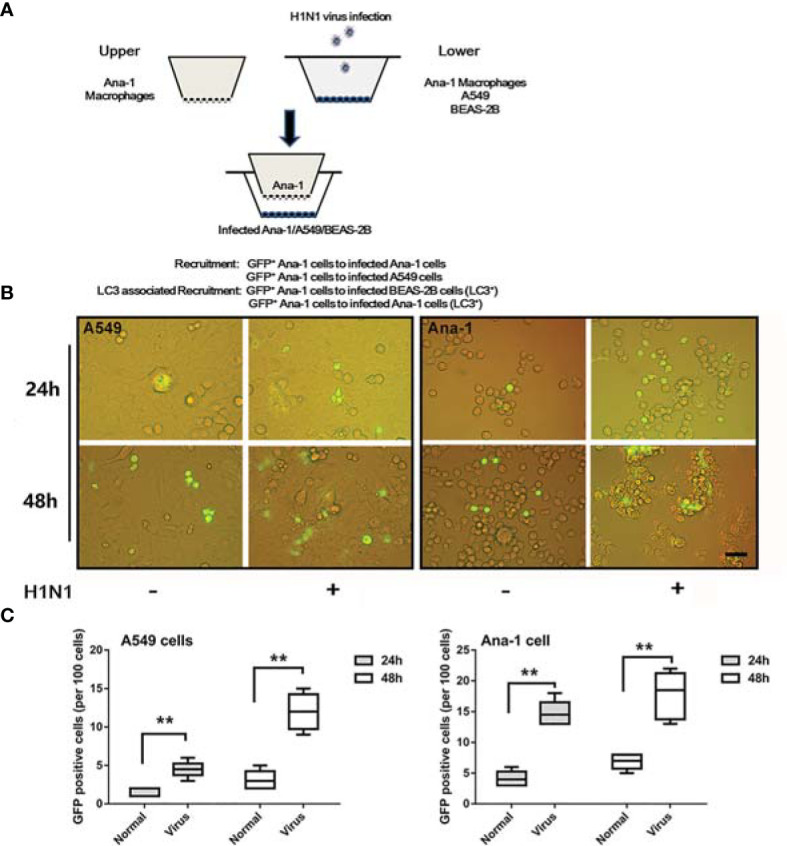
Macrophage recruitment in *in-vitro* infection experiments. **(A)** A systematic study of macrophage recruitment using 24-well transwell chambers (8.0-μM membrane). **(B)** GFP^+^ ANA-1 recruitment in transwell assays. GFP^+^ macrophages were inoculated into the upper chambers, while A549 cells or ANA-1 macrophages were inoculated into the lower chambers and infected with influenza PR/8/34. Scale bar: 30 μm. **(C)** The GFP^+^ ANA-1 macrophages in the lower chambers were enumerated to evaluate the extent of trans-membrane recruitment of macrophages. No fewer than three fluorescent images were captured and analyzed per group. **p < 0.01.

A cell-to-cell transwell assay system was built to explore macrophage recruitment under virus infection conditions (**Figure 2A**). H1N1 virus-infected ANA-1 macrophages or A549 lung epithelial cells were inoculated in the lower chamber, and GFP-labeled ANA-1 macrophages were cultivated in the upper chamber. GFP^+^ macrophages were observed to be recruited to the infected cells (macrophages or A549) in a time-dependent manner upon tracking the migration of GFP^+^ cells (**Figures 2B, C**). Furthermore, the infected cells and recruited macrophages were markedly aggregated at 48 h post-infection.

Additionally, owing to more pronounced agglomerations observed in ANA-1 macrophages (**Figure 2B**), H1N1 infection might trigger more complicated macrophages than those in the A549 cells despite the similarities in the recruitment pattern.

### Influenza Virus Activated the M1 Polarization of Macrophages and Promoted LC3/CD63/IL1β

The phenotype and other cellular responses in the infected ANA-1 macrophages were explored. A higher proportion of CD86^+^ phenotype cells were observed in infected ANA-1 cells compared with the uninfected control cells (**Figure S1**). Subsequently, overall transcriptional analyses of H1N1 replication (M gene), macrophage phenotype marker genes (*iNOS:* M1 polarized, *Arg-1:* M2 polarized), and proinflammatory cytokine genes (*IL-1β* and *TNF-α*) were performed.

**Figure 3:**
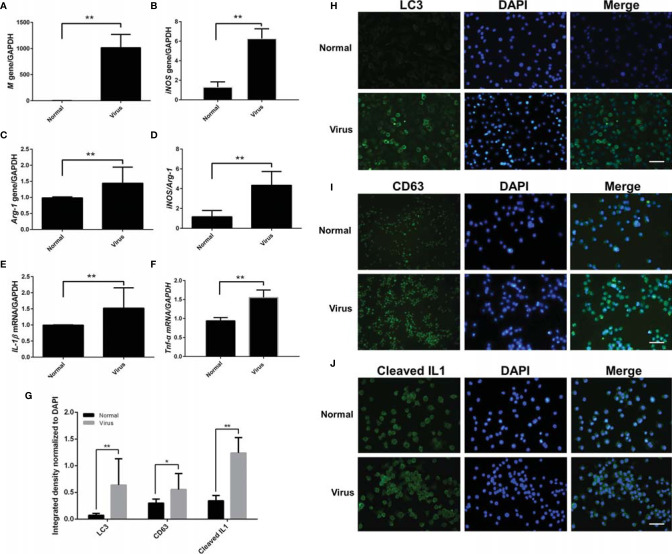
M1 polarization and LC3/CD63/IL-1β analysis of infected ANA-1 macrophages. Total RNA was isolated from infected ANA-1 cells at 24-h post-infection and used for transcriptional analysis of the related genes. **(A)** *Influenza virus M* gene. **(B)** *iNOS* gene (M1 marker). **(C)** *Arg-1* gene (M2 marker). **(D)** *iNOS/Arg-1* (M1/M2 bias). **(E)** *IL-1β* (a proinflammatory cytokine). **(F)** *Tnf-α* (an inflammatory cytokine). N ≥ 3, **p < 0.01. **(G–J)** Normal and infected ANA-1 cells were fixed, incubated with primary antibodies (LC3/CD63/cleaved IL-1β, 1:1000 dilution), and then fluorescently stained with the secondary antibody (anti-rabbit IgG F_ab_2 Alexa Fluor ^®^ 488 molecular probes, 1:2000 dilution). **(G)** The cells’ fluorescence intensity of LC3, CD63, and cleaved IL1-1β was quantified and normalized by the corresponding DAPI fluorescence amount using the Image-Pro Plus 6.0 software. No fewer than three fluorescent images were captured and analyzed for each group. *p < 0.05 and **p < 0.01. **(H)** LC3 cellular immunofluorescence. **(I)** CD63 cellular immunofluorescence. **(J)** Cleaved IL-1β cellular immunofluorescence. Scale bar: 50 μm.

As shown in **Figures 3A–F**, high levels of viral replication (*P* < 0.01, **Figure 3A**) were consistent with significant M1 polarization status (>6-fold *iNOS* and 1.5-fold *Arg-1*, equivalent to 4-fold *iNOS/Arg-1* bias *vs.* the uninfected samples, *P* < 0.01, **Figures 3B–D**) and promoted proinflammatory cytokine transcription (>1.5-fold *IL-1β* and *TNF-α vs.* the normal samples, *P* < 0.01, **Figures 3E, F**).

**Figure 4:**
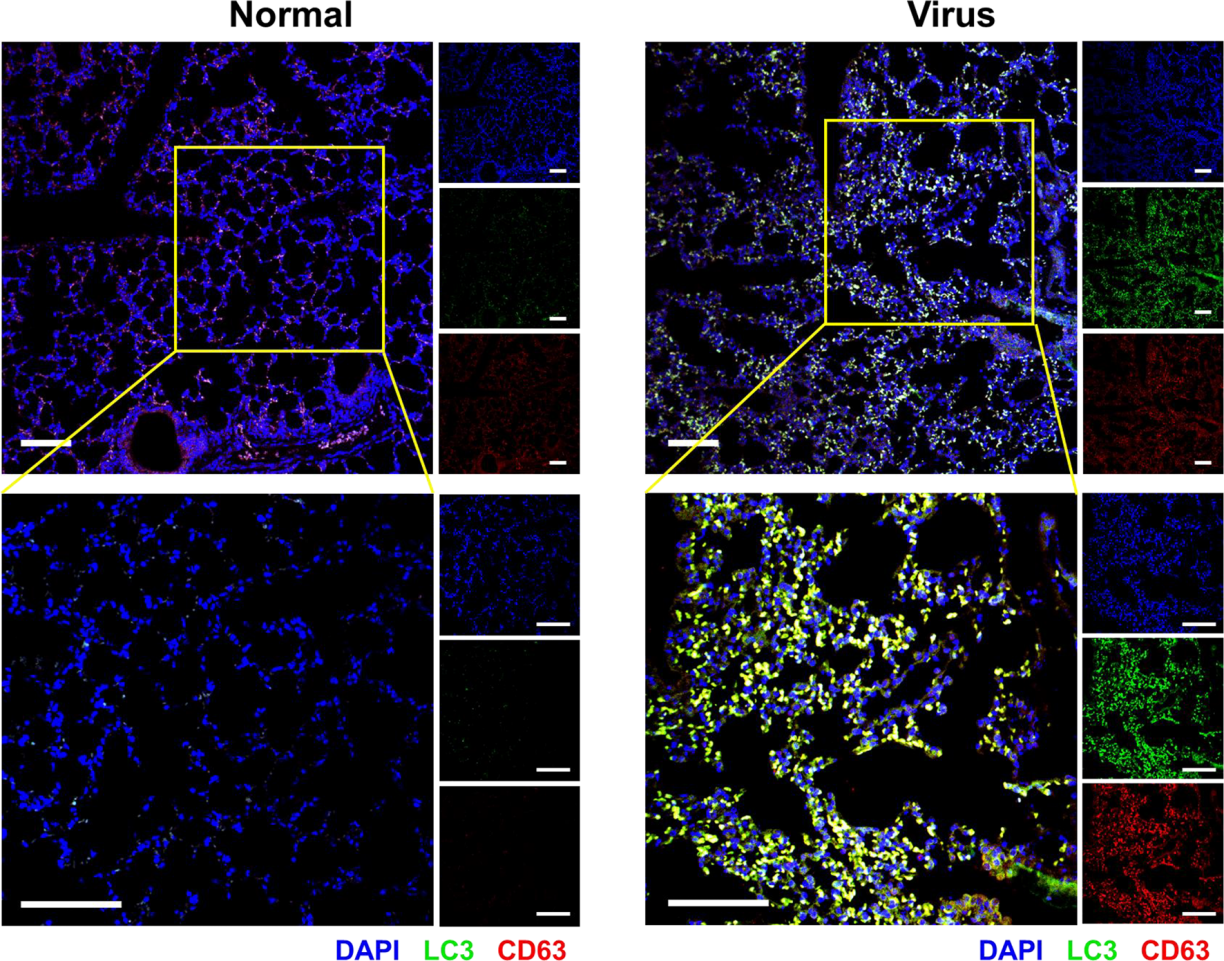
Autophagy and exosomes in the infected lung tissues. Immunofluorescence histochemical staining of the lung tissue to determine the expression of LC3 (green) and CD63 (red) and their colocalization (yellow). The nuclei were stained with DAPI (blue). Scale bar: 100 μm.

Simultaneously, cellular immunofluorescence staining was used to evaluate several possible cell behaviors (LC3: cell autophagy; CD63: exosome; cleaved IL-1β: proinflammatory activation). As shown in **Figures 3G–J**, remarkable upregulation of LC3 and CD63 indicated autophagy, and exosomes were obviously activated by virus infection. IL-1β cleavage denoted the proinflammatory activation of macrophages. The increased expression and colocalization of LC3 and CD63 were also observed in the lung tissues of H1N1-infected mice with immunofluorescence histochemical staining (**Figure 4**).

### Exosomes Possibly Mediated the Recruitment of Macrophages and Their M1 Polarization

**Figure 5:**
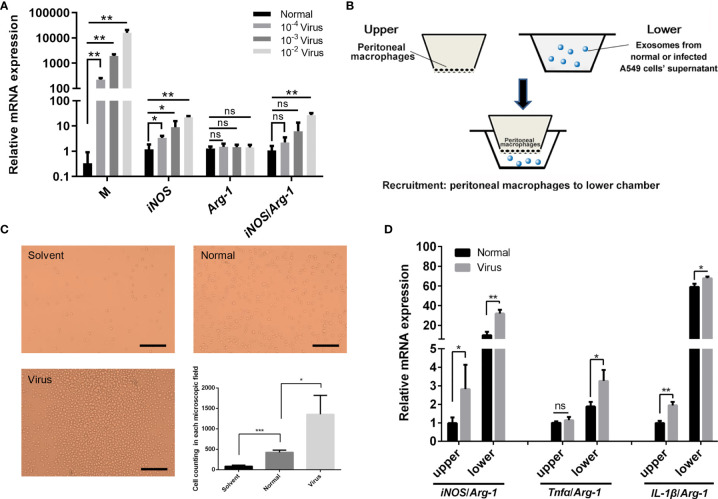
Effects of H1N1 virus infection and exosomes on the polarization and recruitment of peritoneal macrophages. **(A)** Mouse peritoneal macrophages were infected with a gradient dilution (10^-2^, 10^-3^, 10^-4^) of the H1N1 virus, and total RNA was isolated for the transcriptional analyses of *Influenza virus M*, *iNOS, Arg-1 genes.* N = 3, *p < 0.05 and **p < 0.01. **(B)** Schematic diagram of the transwell assay in order to investigate the recruitment of peritoneal macrophages by exosomes. The upper chambers were covered with peritoneal macrophages, and the lower chambers were supplemented with a fresh culture medium (solvent group) or the medium containing exosomes from normal or infected A549 cell’ supernatant (normal or virus groups). **(C)** Cells in the lower chambers were microscopically observed and counted at 24 h after adding exosomes. Scale bar: 50 μm. **(D)** Total RNA was isolated from peritoneal macrophages in the upper and lower chambers at 24 h after adding exosomes and employed for transcriptional analysis of *iNOS*, *Tnfα, IL-1β, Arg-1 genes*. N ≥3, *p < 0.05, **p < 0.01 and ***p < 0.001. ns, no significance.

The exosomes were extracted from the supernatant of the normal or H1N1-infected A549 cells, and the purified exosome samples were characterized, as shown in **Figure S2**. The specific shape of the exosome was observed in both samples by transmission electron microscopy (Hitachi, HT-7700). The mean diameters and concentrations of the exosome samples in the normal and H1N1-infected groups were 76.67 nm and 2.11×10^9^ particles/mL and 83.66 nm and 3.26×10^10^ particles/mL, respectively. Furthermore, the positive rates of exosome CD63 expression were 20.9%. The specific protein markers, CD9, and syntenin (25), were also detected to identify the exosomes. As shown in **Figure 5A**, the M1 polarization of peritoneal macrophages was positively correlated with viral replication in a dose-dependent manner. In transwell experiments, exosomes steered the recruitment of more peritoneal macrophages to the lower chamber (**Figures 5B, C**). Compared with the normal group, the exosomes in the virus-infected group promoted macrophage recruitment to a greater extent (**Figure 5C**). The phenotypes of the peritoneal macrophages in the upper and lower chambers were analyzed. The exosomes of the infected A549 cells were found to enhance M1 polarization (*iNOS*/*Arg-1, Tnfα*/*Arg-1*, and *IL-1β*/*Arg-1*) in a better way (**Figure 5D**), thereby indicating that exosomes might play an essential role in influenza-mediated macrophage polarization. To exclude the possible interference of residual viruses in the exosomes, the expressions of the M gene in ANA-1 cells directly infected by H1N1 and ANA-1 cells supplemented with exosomes derived from normal or H1N1 infected cells were detected (**Figure S3**). The M gene level represents the relative amounts of the H1N1 virus. Compared with cells directly infected by H1N1, few viruses were detected in exosome-supplemented macrophages, which implied the presence of few virus particles in the exosomes. However, these exosomes could still promote M1 polarization of the macrophages, thereby suggesting that the stimulation of polarization in the exosome groups might be caused by exosomes themselves rather than the H1N1 virus. The results in **Figure S3** also showed that macrophages were more M1 polarized by virus-derived exosomes than by exosomes derived from normal cells, which signified that exosomes produced during viral infection could promote polarization of macrophages to a greater extent. The possible role of remaining virions would be figured out in our future experiments.

### Autophagy and Exosome Production Coordinately Enhanced M1 Polarization and Recruitment of the Macrophages

Non-cancerous cell lines (ANA-1 macrophages and BEAS-2B epithelial cells) were further chosen to explore the role of autophagy by introducing mRFP-LC3 lentiviral vectors to overexpress LC3 labeled with a red fluorescent protein (RFP).

**Figure 6:**
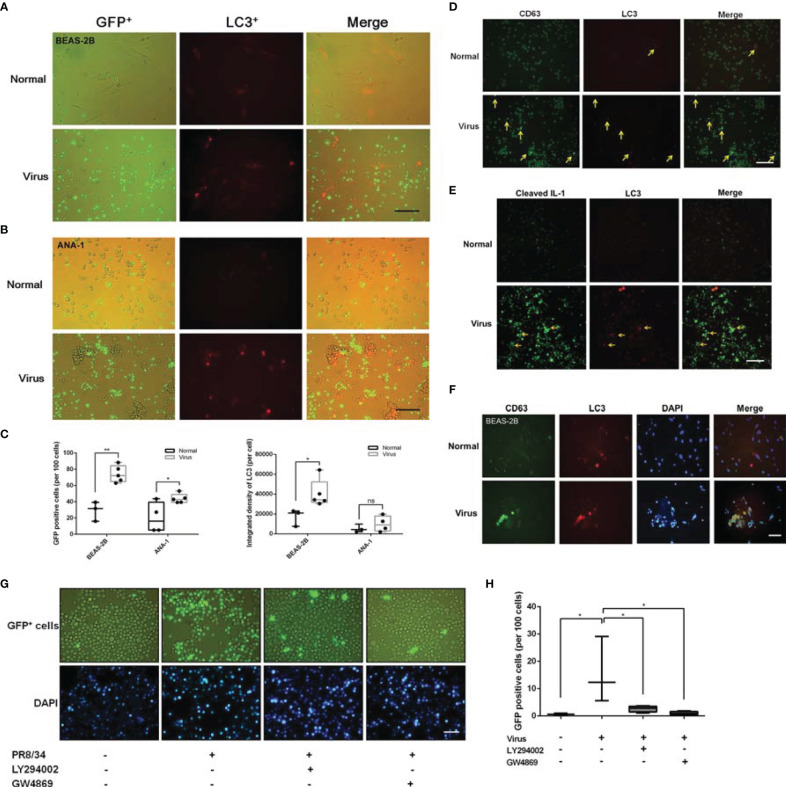
Crosstalk among LC3, CD63, and cleaved IL-1β in macrophage recruitment. **(A, B)** Transwell assays results showed that LC3 enhanced macrophage recruitment in PR/8/34 infected BEAS-2B cells **(A)** and ANA-1 cells **(B)**. GFP^+^ ANA-1 cells were incubated in the upper chambers, BEAS-2B and ANA-1 cells transfected with mRFP-LC3 lentivirus were incubated in the lower chambers and infected with PR/8/34 virus. Scale bar: 100 μm. **(C)** Cell counting of GFP-positive cells (per 100 cells) and the fluorescent quantitation of LC3 (per cell) using the Image-Pro Plus 6.0 software. No fewer than three fluorescent images were captured and analyzed per group. *p < 0.05 and **p < 0.01. **(D, E)** LC3, CD63, and IL-1β interaction. The ANA-1 cells transfected with mRFP-LC3 lentivirus were infected with PR/8/34 virus for 24 h. Then, these cells were fixed, permeabilized, blocked, probed with primary antibodies for the detection of CD63 **(D)**, and cleaved IL-1β **(E)**, followed by exposure to the anti-rabbit IgG Fab2 Alexa Fluor ^®^ 488 molecular probes. After staining the cell nuclei with DAPI, the interaction between LC3/CD63/IL-1β was observed and imaged. Scale bar: 100 μm. **(F)** LC3 and CD63 interaction in the infected BEAS-2B cells. The BEAS-2B cells transfected with mRFP-LC3 lentivirus were infected with PR/8/34 virus for 24 h. The cells were then fixed, permeabilized, blocked, and probed with primary antibodies for the detection of CD63, followed by the anti-rabbit IgG Fab2 Alexa Fluor^®^ 488 molecular probes. After staining the cell nuclei with DAPI, the interaction of LC3/CD63 was observed and imaged. Scale bar: 50 μm. **(G)** The influence of autophagy and exosome inhibitors on macrophage recruitment. GFP^+^ ANA-1 cells were incubated in the upper chambers of the transwell system, while ANA-1 cells were incubated in the lower chambers, infected with PR/8/34 virus, and treated with the autophagy inhibitor LY294002 (1 µM) and the exosome inhibitor GW4869 (1 µM). GFP^+^ ANA-1 macrophages in the lower chambers were counted to evaluate the trans-membrane recruitment of macrophages. Scale bar: 50 μm. **(H)** Cell counting of GFP-positive cells (per 100 cells). No fewer than three fluorescent images were captured and analyzed per group. *p < 0.05. ns, no significance.

Tracking the labeled cells revealed that GFP^+^ ANA-1 macrophage recruitment was positively correlated with LC3 augmentation and increased aggregation of GFP^+^ ANA-1 with infected BEAS-2B cells at 24 h post-infection (**Figures 6A, C**). As shown in **Figures 6B, C**, similar recruitment of GFP^+^ ANA-1 macrophages was observed in the infected macrophages. This finding suggested that upregulated autophagy by H1N1 enhanced the recruitment of remote macrophages.

Based on the role of autophagy and exosome production in macrophage recruitment and M1 polarization, whether autophagy and exosome production coordinately regulated the activation of macrophages was determined. As shown in **Figure 6D**, remarkable upregulation of LC3 and CD63 was observed in the infected macrophages. Apparent overlaps in fluorescence (yellow arrow) were observed in the margined LC3 (red) and CD63 (green) fluorescence images, which suggested a possible and tight interaction between autophagy and exosome production.

The correlation between autophagy and macrophage proinflammatory activation was investigated. As shown in **Figure 6E**, there was some overlap between the fluorescence images of cleaved IL-1β^+^ cells and LC3^+^ cells, and a large proportion of the cleaved IL-1β^+^ cells were aggregated with the LC3^+^ cells, which indicated that autophagy steered macrophage recruitment and activation.

The overlap between LC3 and CD63 in infected BEAS-2B cells (**Figure 6F**) and lung tissues (**Figure 4**) verified the possible interaction between autophagy and exosome production.

These aspects were further explored in infected primary peritoneal macrophages using western blotting. As shown in **Figure S4**, LC3 and CD63 decreased in a similar manner and were accompanied by the accumulation of the autophagy substrate p62 and the augmentation of cleaved IL-1β. This evidence indicated that the canonical autophagic flow was blocked in the infected cells and that LC3 protein likely enhanced exosome production and secretion.

When the infected ANA-1 macrophages (lower chambers in transwell experiments) were treated with the autophagy inhibitor LY294002 (1 µM) and the exosome inhibitor GW4869 (1 µM), the recruitment of GFP^+^ macrophages was significantly reduced (**Figures 6G, H**).

**Figure 7:**
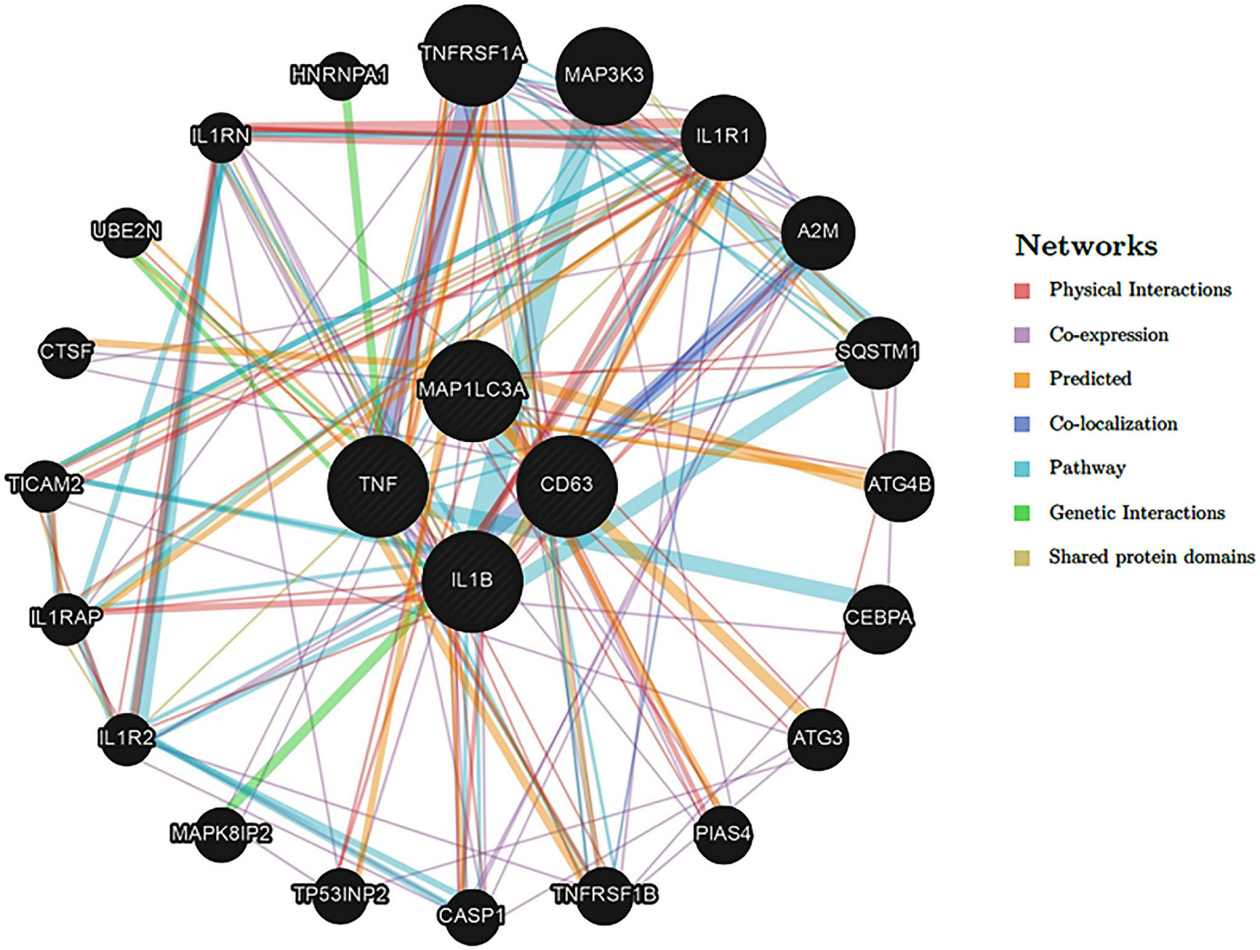
Network analysis of autophagy and exosome genes. The possible interactions of the related genes were predicted using the GeneMANIA database (http://genemania.org/).

The possible interaction between these functional proteins (LC3 and CD63) was also supported by GeneMANIA Bank (https://genemania.org) network analysis. As shown in **Figure 7**, possible coexpression of the genes involved in autophagy and exosome production was found.

Thus, a possible and sequential activation of macrophages triggered by H1N1 infection in autophagy and exosome-dependent manner was established.

## Discussion

This study revealed that autophagy and exosome production are involved in H1N1-induced macrophage recruitment and M1 polarization.

Many researchers have reported that inhibiting overactive immune responses can offer protection against H1N1-induced severe viral pneumonia (20, 26–28). However, the detailed interactions among the influenza virus, immune cells, and epithelial cells remain unclear. In this study, M1 polarized macrophage recruitment was observed to be possibly responsible for the exacerbated inflammatory responses and high mortality of the infected mice. *In vitro*, the transwell experiment system was successfully employed to mimic the recruitment and M1 polarization of the macrophages, which provided a straightforward method to explore the possible crosstalk among the different cells involved in the process of virus infection.

Macrophages play an essential role in influenza virus infection. During the infection, alveolar macrophages, as well as neutrophils, perform the function of phagocytizing apoptotic infected cells, thereby arresting viral propagation (29). Alveolar macrophages differ in their susceptibilities to different viral strains. Strains with weak infectivity toward macrophages, such as PR8 (H1N1), exhibit high virulence in mice, perhaps because macrophages are not activated directly by the virus. IAV strains that infect macrophages, such as ST169 (H1N1) and ST602 (H3N2), can stimulate the M1 polarization of macrophages, a phenotype with highly expressed iNOS and proinflammatory cytokines. M1 macrophages also possess enhanced phagocytic ability and are capable of eliminating intracellular pathogens (30). In our study, murine macrophages (ANA-1) and primary peritoneal macrophages were infected by A/PR/8/34 (H1N1) and polarized to M1 phenotype with the secretion of several proinflammatory cytokines, thus inducing the polarization and recruitment of more macrophages.

In previous studies, cell autophagy has been proven in influenza virus replication and viral pathology (31–34), and inhibiting autophagy has been shown to suppress H3N2 replication *in vitro* (35). In this study, activation of autophagy (LC3 upregulation) was found to correspond to H1N1 infection and correlated positively with macrophage recruitment. Furthermore, LC3 seemed to induce marked cell infusion (abnormal morphology) and promote macrophage aggregation in response to infection. Thus, autophagy might result in morphological abnormalities in the infected cells and promote macrophage phagocytosis. These results were partly confirmed by other studies, thereby asserting that atypical autophagy induces LC3-associated phagocytosis (36–38). However, the role of autophagy-associated phagocytosis in H1N1 infection needs to be elucidated in the future *via* an appropriate experimental design. In addition, autophagy can be classified into canonical and noncanonical, both of which are marked by LC3; however, which one plays a dominant role in IAV infection is still obscure. Studies have shown that noncanonical autophagy reduces IAV replication and fusion with endosome and inhibits interferon signaling, which helps control lung inflammation (39). Therefore, further studies are needed to distinguish between canonical and noncanonical autophagy.

Exosomes are extracellular vesicles measuring 30-150 nm and are released by almost all cell types, including stem cells (40) and cancer cells. Exosomes are significantly altered in many diseases and physiological states (41–44). In this study, significant upregulation of CD63 (exosome marker) was found in the virus-infected cells. Moreover, exosome production and autophagy pathways exhibited synergies in cellular homeostasis and metastasis (45, 46). Also, an overlap of LC3 and CD63 immunofluorescence imaging was noted in H1N1-infected cells. Some reports have shown that LC3 participates in the production of exosomes known as autophagic exosomes, which function *via* a mechanism that is independent of canonical macroautophagy (47–49). Autophagosomes can fuse with multivesicular bodies to form amphisomes (50), which in turn fuse with the plasma membrane. The intraluminal vesicles present in the amphisome are released from the cell as exosomes, which carry both viral nucleic acids and proteins and transmit immune signals between cells (51). Employing the transwell method, exosomes from the supernatant of H1N1-infected A549 cells were demonstrated to induce the recruitment and M1 polarization of peritoneal macrophages. This phenomenon was more remarkable than that in the noninfected group and the solvent group. When autophagy and exosome production in the infected macrophages were inhibited with LY294002 and GW4869, the recruitment of GFP+ macrophages was observed to dramatically decrease in a pattern that was quite similar to the one observed in normal cells. These findings indicate that autophagy and exosome production coordinately enhance macrophage recruitment, although the detailed interactions are yet to be clarified.

Influenza virus infection induces a severe cytokine storm that releases several proinflammatory cytokines, such as TNFα, IL-1β, IL-6, and IFN-γ. Pro-IL-1β is upregulated by activated NF-κB and then matures into cleaved IL-1β and is secreted under the influence of NOD-like receptor protein 3 (NLRP3). IL-1β could recruit neutrophils and T cells and induce the epithelial and endothelial cells to produce TNF-α and IL-6, thus aggravating lung inflammation and injury (52). In this study, a cleaved IL-1β antibody was used to label the macrophages, and the possible correlation between autophagy and macrophage proinflammatory activation was explored. Some overlap was seen in the immunofluorescence signals of cleaved IL-1β and LC3, but more IL-1β^+^ cells were found to be aggregated with the LC3^+^ cells (**Figure 6E**), which suggests that upregulated autophagy enhances the activation of M1 macrophages.

The exosomes were also purified using 70-nm qEV2 size-exclusion chromatography (SEC) columns and characterized by transmission electron microscopy, the particle diameter, and exosomes markers analysis (NanoFCM and Western blotting). The purified exosome sample from virus-infected cells showed more significant M1 stimulation on macrophages and minor virus replication. However, the possible role of remaining virions would be designed in our future experiments.

Based on these results, we propose that autophagy and exosome production coordinately induce M1 polarization and recruitment of the macrophages *via* a possible autophagic exosome pathway. Exosomes serve as a vehicle for transporting cargo from parental cells to recipient cells. However, the components of the vesicles (LC3, CD63, virus-related proteins, DNA strands, mRNA, microRNAs, lncRNAs, and circRNAs) responsible for these effects have not yet been identified (53–56).

This study on autophagy and exosome production in pulmonary cells may shed light on potential therapeutic strategies to manage respiratory diseases caused by the influenza virus and other such viruses. For instance, mesenchymal stem cell-derived exosomes have been proven to have the potential to cure SARS-CoV-2 pneumonia owing to their anti-inflammatory effects and immune-modulating capabilities (57, 58). Some modulators of autophagy or exosome production may also be used to intervene in disease processes (59) and have implications in the treatment of influenza.

Above all, our findings might provide feasible targets for treating influenza virus infection by interfering with macrophage activation and recruitment.

## Data Availability Statement

The raw data supporting the conclusions of this article will be made available by the authors, without undue reservation.

## Ethics Statement

The animal study was reviewed and approved by The Animal Experiment Committee of Fudan University.

## Author Contributions

XS designed the study. CX, WX, YZ, and XA performed the experiments. CX, PG, and YN collected the samples, analyzed the data, and wrote the manuscript. HZ, WZ, and HH contributed new analytical tools or models. All authors contributed to the article and approved the submitted version.

## Funding

This work was funded by a grant from Nation Nature Science Foundation of China (grant number: 82074097), Key Program of Nation Nature Science Foundation of China (grant number: 82030113 and U1604283), Shanghai Science and Technology Funds (grant number: 17ZR1401700), and Shanghai Municipal Health Commission project (grant number: 201740200).

## Conflict of Interest

The authors declare that the research was conducted in the absence of any commercial or financial relationships that could be construed as a potential conflict of interest.

## Publisher’s Note

All claims expressed in this article are solely those of the authors and do not necessarily represent those of their affiliated organizations, or those of the publisher, the editors and the reviewers. Any product that may be evaluated in this article, or claim that may be made by its manufacturer, is not guaranteed or endorsed by the publisher.
