# Supplementary material for: Autophagy and Exosome Coordinately Enhance Macrophage M1 Polarization and Recruitment in Influenza A Virus Infection
Source: Front Immunol. 2022 Mar 17;13:722053. doi: 10.3389/fimmu.2022.722053 (PMC8967985; doi:10.3389/fimmu.2022.722053)
Supplement: Supplementary file 1 [file DataSheet_1.docx]

Fig. S1


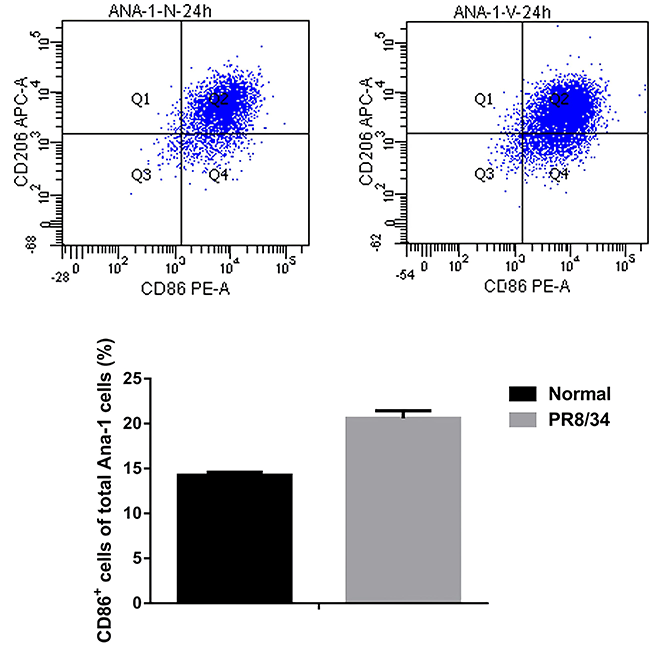


Murine Ana-1 macrophages were infected with 10TCID_50_of H1N1 virus (PR/8/34, ATCC) for 2 h and then re-cultivated in a fresh medium for 24 h. The harvested cells were stained with fluorescence-labeled antibodies against CD86^+^ (M1 phenotype) and CD206^+^ (M2 phenotype). Non-infected Ana-1 cells were set as the normal sample.

Fig. S2


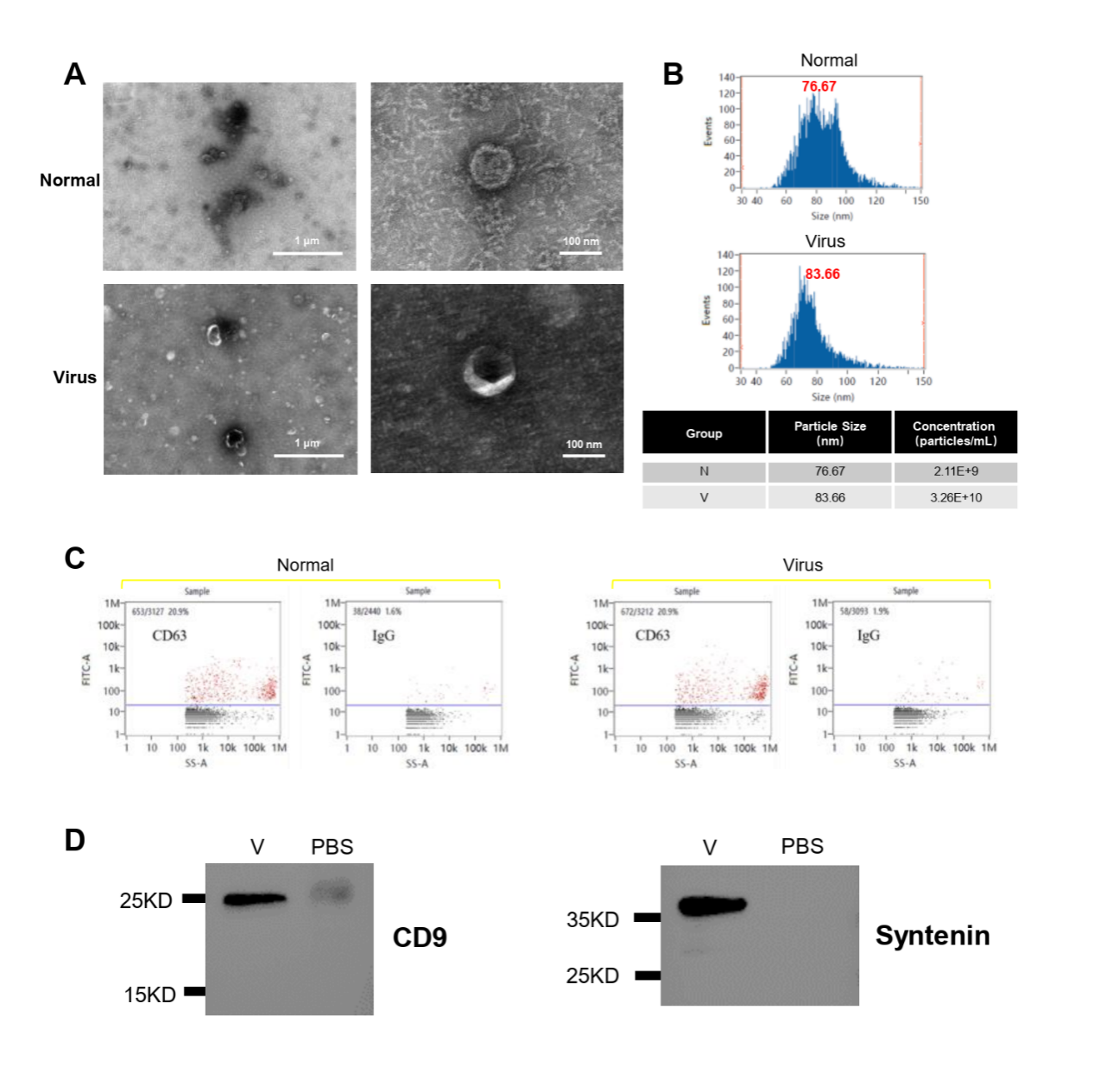


Characterization analysis of exosomes extracted from the supernatant of normal or H1N1 virus-infected A549 cells. (A) The exosome samples observed by transmission electron microscopy (Hitachi, HT-7700), (B) and the diameters and the concentration of exosome samples were measured by using a particle size analyzer (NanoFCM, N30E). The red numbers in the pictures depict the average particle size. (C) Exosome samples were incubated with FITC-labeled antibodies against CD63 or IgG and detected by NanoFCM. (D) For exosomes extracted from the supernatant of H1N1 virus-infected A549 cells, the expression of CD9 and syntenin was detected by Western blotting. PBS was used as the negative control.

Fig. S3


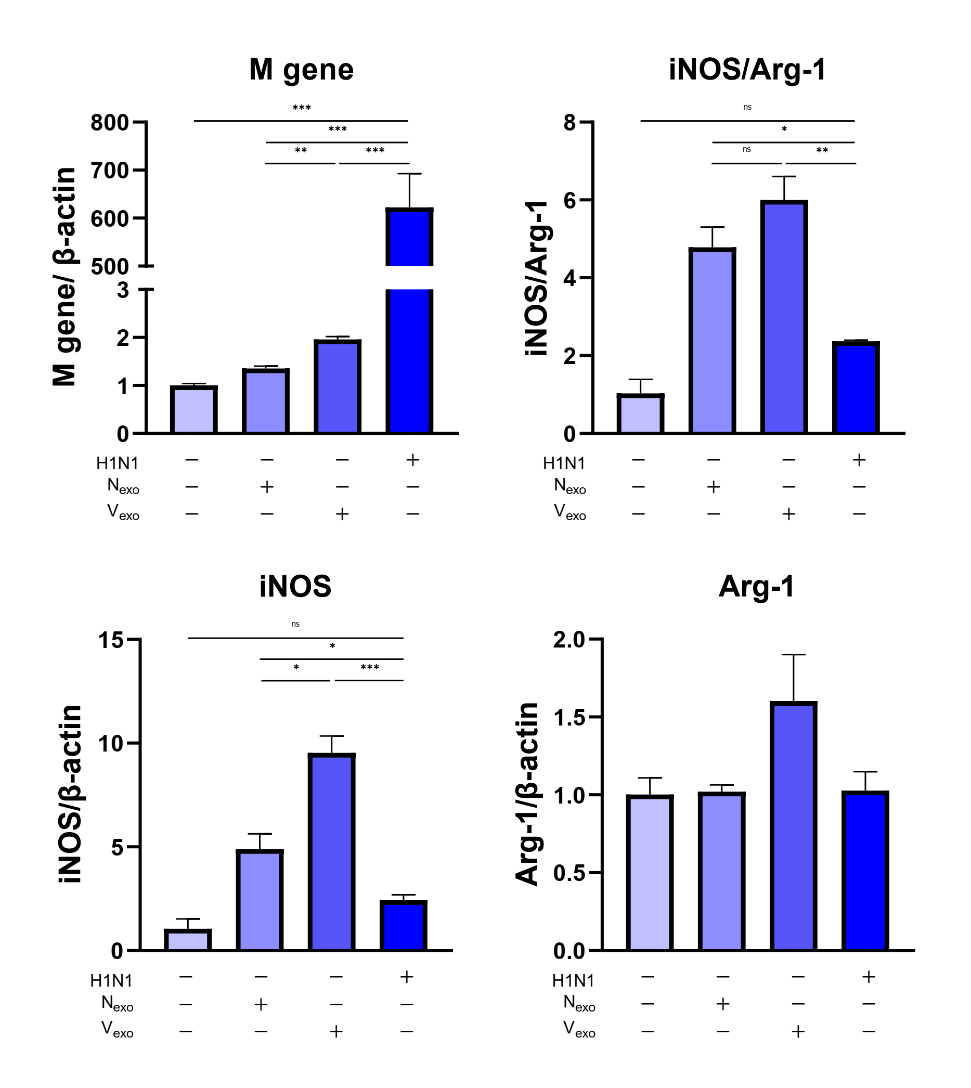


Exosomes were extracted from normal or H1N1-infected A549 cells and added to the culture medium of ANA-1 macrophages for 24 h. ANA-1 macrophages infected with H1N1 virus for 24 h were served as a positive control group. Total RNA was isolated for transcriptional analysis of *Influenza virus M*, *iNOS*, *Arg-1, β-actin* genes. N ≥ 3, *p < 0.05, **p < 0.01 and ***p < 0.001.

Fig. S4


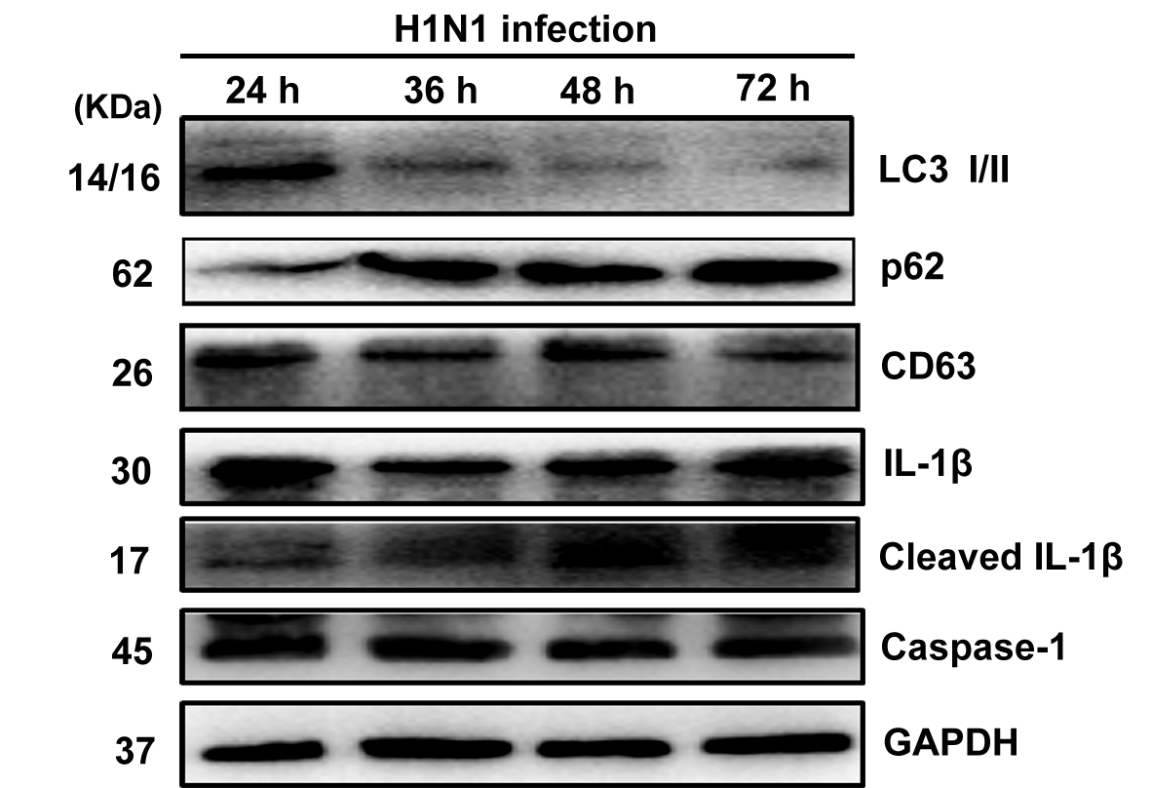


The total proteins of infected primary peritoneal macrophages were extracted at 24, 36, 48, and 72 h post-infection and then subjected for Western blotting. The corresponding antibodies were used to analyze autophagic protein (LC3, p62), exosome marker (CD63), and IL-1β activation pathway (IL-1β, cleaved IL-1β, and caspase-1) normalized to GAPDH.
